# Trajectories of cognitive function development and predictive factors in disabled middle-aged and older adults

**DOI:** 10.3389/fpubh.2024.1436722

**Published:** 2024-09-09

**Authors:** Jiaxue Pang, Yang Xu, Qiankun Liu, Juju Huang, Pengyao Li, Li Ma, Chunlu Zeng, Xiaoqing Ma, Hui Xie

**Affiliations:** College of Nursing, Bengbu Medical University, Bengbu, Anhui, China

**Keywords:** cognitive function, disability, middle-aged and older adults, latent growth mixture models, trajectories

## Abstract

**Objective:**

To explore the trajectories of cognitive function development and predictive factors in disabled middle-aged and older adults.

**Methods:**

Utilizing data from 983 disabled middle-aged and older adults in the China Health and Retirement Longitudinal Study (CHARLS) from 2013 to 2020, latent growth mixture models were constructed to analyze the categories of cognitive function development trajectories and their predictive factors.

**Results:**

The cognitive function trajectories of the disabled middle-aged and older adults were classified into three categories: rapid decline (32.6%), Slow decline (36.1%), and Stable (31.2%). Multinomial logistic regression analysis identified age, gender, residence, education, marital status, household income, sleep duration, depression, hearing ability, and social participation as predictors of these trajectories.

**Conclusion:**

There is heterogeneity in the cognitive function development trajectories among disabled middle-aged and older adults. Healthcare professionals can implement targeted health management based on the characteristics of different groups to prevent the deterioration of cognitive function in this population.

## 1 Introduction

Cognitive function is an important indicator reflecting health status, directly influencing an individual’s ability to perform daily activities and overall quality of life (1). Cognitive impairment is relatively common among middle-aged and older adults. It is known that cognitive impairment is an early clinical symptom of dementia, which may later develop into cognitive disorders, Alzheimer’s disease, and other conditions (2). Currently, approximately 10 million older adults in China suffer from cognitive disorders, accounting for one-fifth of the world’s population with cognitive disorders. This number is projected to increase to 16 million by 2030 (3). There are currently no effective treatments for cognitive impairment or dementia (4). Therefore, identifying modifiable risk factors associated with cognitive decline is crucial for delaying and preventing the onset of cognitive impairment and/or dementia.

According to the 2006 Second National Sample Survey on Disability in China, older adults with disabilities constitute 24.43% of the total older population and 53.24% of the total disabled population (5). Recent statistics indicate that individuals aged 45 and above account for 43.43% of the population, with those aged 60 and above comprising 18.94% (6). With the intensification of population aging, the number of middle-aged and older adult individuals with disabilities is gradually increasing. There is a close association between disability and cognitive impairment. Research indicates that disability and cognitive impairment often co-occur, sharing common risk factors and biological mechanisms, including disease-dependent and age-dependent mechanisms (7). From 2010 to 2020, the proportion of older adults with cognitive impairment in China increased from 13.26 to 18.7% (3). Another study based on national survey data revealed that the detection rate of cognitive impairment among older adults with disabilities in China is as high as 56.13% (8), significantly higher than the overall detection rate of cognitive impairment among the older adults. The cognitive function status of the older adults disabled population is therefore a cause for concern.

Currently, research on depression among middle-aged and older adults with disabilities is noticeably lacking. To our knowledge, no studies have been conducted on the trajectories of cognitive function changes in disabled middle-aged and older adults. Previous research on the relationship between cognitive function and disability has mostly been based on cross-sectional surveys (9, 10), which do not account for the dynamic nature of cognitive function, influenced by health status, lifestyle, and environmental factors over time. Moreover, cognitive changes in middle-aged and older adults follow different differentiated trajectories, exhibiting heterogeneity (11, 12). Therefore, it is essential to enhance in-depth research on cognitive function among the disabled middle-aged and older adults to promote a comprehensive understanding of their cognitive status and to prevent or delay the onset of cognitive impairment in this demographic.

The health ecology model applies ecological theory and methods to the health domain, emphasizing the multi-layered influence of the social environment on individuals. This model posits that individual health results from the interplay of individual factors, social environment, healthcare policies, cultural customs, and other elements (13). In recent years, the health ecology model has been widely applied in the medical and health fields. Utilizing this model allows for a multidimensional exploration of the cognitive function trajectories and their predictive factors among disabled middle-aged and older adults, thereby providing new approaches and perspectives for the prevention and control of cognitive function decline in this population (14). Previous studies adopting this health ecological perspective have demonstrated that the factors influencing cognitive function are multifaceted (15).

Based on this model, this study utilizes four waves of data from the China Health and Retirement Longitudinal Study (CHARLS) to conduct a comprehensive analysis of the cognitive function trajectories, considering five factors ranging from individual characteristics to external environment. The aim is to analyze the cognitive function changes and their predictive factors among the disabled middle-aged and older adults in China, providing a theoretical basis for identifying and intervening in cognitive impairment in this demographic, thereby promoting healthy aging.

## 2 Materials and methods

### 2.1 Data collection

The China Health and Retirement Longitudinal Study (CHARLS) is a large-scale, interdisciplinary survey project hosted by the National School of Development at Peking University. It collects high-quality longitudinal survey data through household interviews from a nationally representative sample of individuals aged 45 and above and their spouses. The survey covers 150 counties and 450 communities (villages) across 28 provinces (autonomous regions and municipalities) in China. The baseline survey was conducted in 2011, with follow-up assessments every 2 years, including physical measurements during each follow-up. The CHARLS database comprises seven modules: basic demographic information, family information, health status and functioning, healthcare and insurance, work, retirement and pension, income, expenditure and assets, and housing. This study has received approval from the Biomedical Ethics Review Committee of Peking University (Ethics approval number: No. IRB00001052-11015), and all participants have provided informed consent.

**Figure 1:**
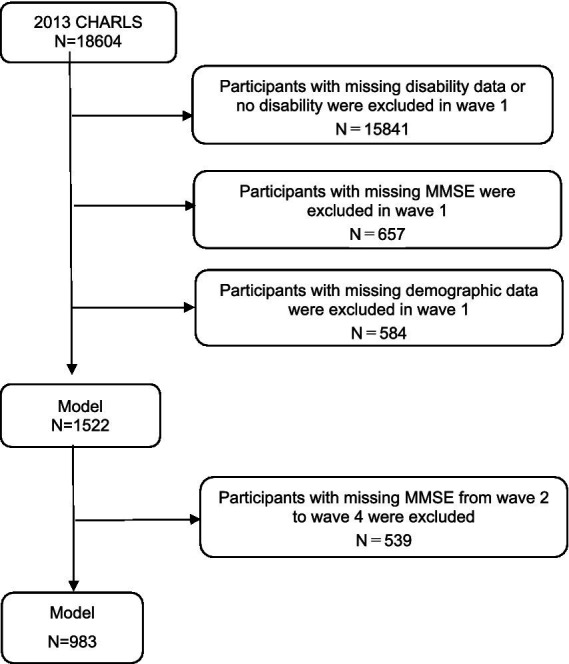
CHARLS selection process flowchart.

This study utilized four waves of CHARLS data from 2013, 2015, 2018, and 2020. Based on the research objectives, the inclusion criteria were: age ≥ 45 years in the 2013 baseline survey; ADL scale (14) includes six activities: dressing, bathing, eating, getting in or out of bed, using the toilet, and controlling bowel and bladder functions, where difficulty in completing any one of these tasks is defined as ADL functional limitation (disability) (16); full participation in all four waves of the survey; complete demographic information, required health-related information, physical examination data, etc. The exclusion criterion was unwillingness to participate in the CHARLS survey. In this longitudinal cohort study, a total of 983 participants were selected, excluding those under 45 years of age, non-disabled, lost to follow-up, and those with missing cognitive function data. The response rates for waves 1 to 4 were 88.3, 87.1, 86.4, and 86.8% (17). The complete process of participant selection is shown in Figure 1.

### 2.2 Cognitive function

**Figure 2:**
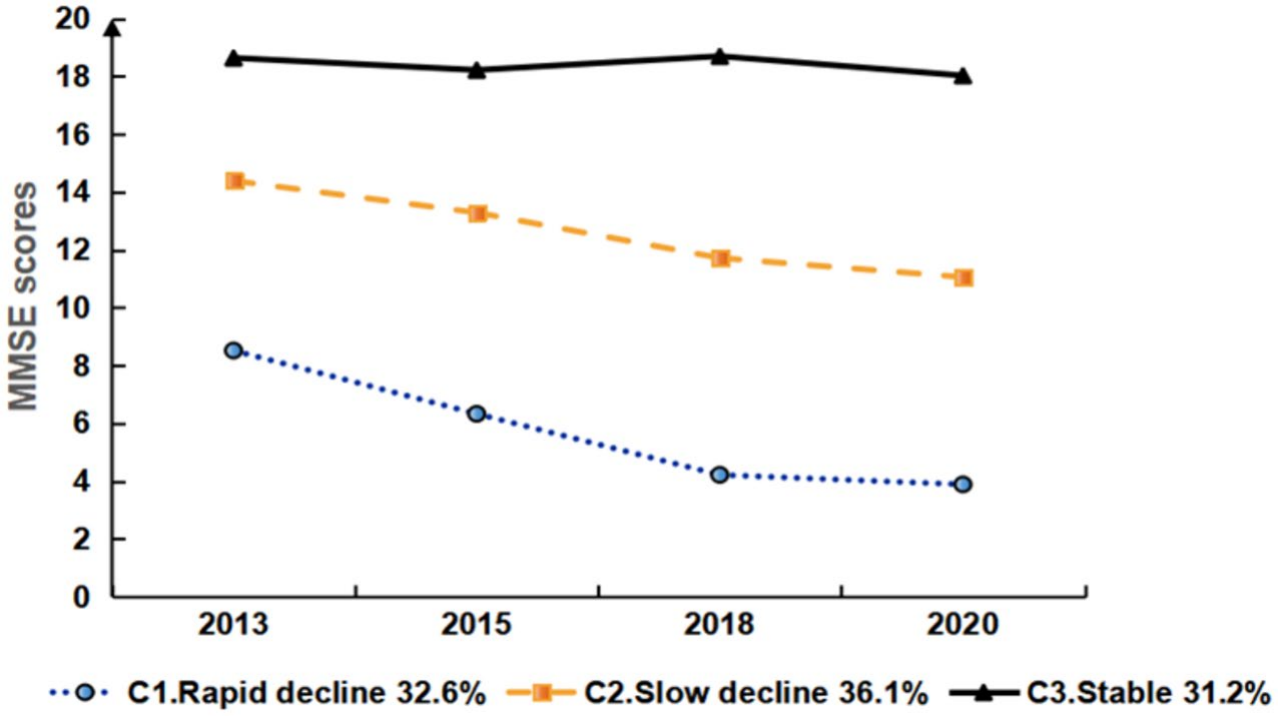
Development trajectory of cognitive function in disabled middle-aged and older adult people.

Cognitive function scores are calculated using the Telephone Interview for Cognitive Status (TICS) from the CHARLS questionnaire, encompassing three dimensions: mental status, immediate recall ability, and delayed recall ability. Mental status involves asking the respondent about the current year, month, date, day of the week, and season, and to subtract 7 sequentially five times from 100, with the number of correct answers contributing to the score, up to 10 points. Immediate recall ability involves reading 10 words to the respondent and scoring the number of words correctly recalled, also up to 10 points. Delayed recall ability tests recall of the same 10 words after a time interval, with each correct word also scoring a point, up to 10 points. The total possible score is 30 points, with higher scores indicating better cognitive function (17). Cognitive impairment was defined according to the educational level of the participants: illiterate individuals scoring <17, primary school education <20, secondary school education (including vocational school) < 22, and college education (including junior college) < 23 (18) (Figure 2).

### 2.3 Variable

The health ecology model is one of the derivative models of ecology and has been widely applied to analyze the influencing factors of individual diseases or health (19). This model emphasizes that an individual’s health is the result of the combined effects of the individual and the environment. It advocates analyzing the influencing factors of health or disease from multiple levels, including the individual and the environment, to provide health information from different dimensions and ultimately promote health (20). The health ecology model is mainly divided into five dimensions:

Personal characteristics: Variables include age (1 = 45–59 years, 2 = 60 years and above), gender (1 = male, 2 = female), and chronic diseases (1 = yes, 2 = no);Behavioral characteristics: Variables include smoking (1 = yes, 2 = no), sleep duration (1 = <6 h, 2 = 6–8 h, 3 = >8 h), self-rated health (1 = good, 2 = average, 3 = poor), and hearing ability (1 = good, 2 = average, 3 = poor). Interpersonal network: Variables include marital status (1 = married, 2 = not married), residence (1 = rural, 2 = urban), social participation (1 = yes, 2 = no), and depression (1 = yes, 2 = no). Living and working conditions: Variables include education level (1 = illiterate, 2 = primary school and below, 3 = junior high school and above) and family income (1 = annual income <15,000 RMB, 2 = 15,000–30,000 RMB, 3 = >30,000 RMB). Policy environment: Variables include medical insurance (1 = yes, 2 = no) and pension insurance (1 = yes, 2 = no).

Definitions of some independent variables are as follows: (1) Smoking: Defined as having smoked in the past year; (2) Social participation: Assessed by the question “Have you participated in the following social activities in the past month?” Participation in at least one social activity is considered as social participation; otherwise, it is considered as no participation; (3) Hearing ability: Measured by the question “How is your hearing?” with answers including “excellent, very good, good, average, poor.” In this study, “excellent, very good, good” are categorized as good, “average” as average, and “poor” as poor; and (4) Pension insurance: Assessed by the question “Are you currently participating in or receiving any of the following pension insurances?” Participation in at least one is considered as having pension insurance; otherwise, it is considered as not having pension insurance.

### 2.4 Statistical analysis

Statistical analyses were conducted using Mplus version 8.3 software to perform latent growth mixture modeling (LGMM). The number of classes was incrementally increased, and model fit indices were compared to determine the optimal model based on practical significance and statistical indicators. Key fit indices included the Akaike Information Criterion (AIC), Bayesian Information Criterion (BIC), and sample-size adjusted BIC (aBIC), with lower values indicating better model fit. Entropy, ranging from 0 to 1, was also considered. Model comparison utilized the Lo–Mendell–Rubin (LMR) test and the Bootstrap Likelihood Ratio Test (BLRT). A *p*-value less than 0.05 indicated that the fit of model K was superior to that of model K-1. Descriptive analysis was carried out using SPSS version 26.0, presenting count data as frequencies or percentages, and normally distributed metric data as means ± standard deviations (x ± s). Multivariate logistic regression was employed to analyze predictive factors for cognitive trajectories among the disabled middle-aged and older adults, with a significance level set at α = 0.05.

The baseline data from the 2013 CHARLS survey included variables across five dimensions: personal characteristics, behavioral traits, interpersonal networks, work and living environment, and policy environment. These variables were used to explore the predictive factors for changes in cognitive function trajectories among disabled middle-aged and older adults from 2013 to 2020.

## 3 Results

### 3.1 Descriptive characteristics

At the 2013 baseline, the study included 983 disabled older adult participants with an average age of 61.10 ± 8.71 years. The majority were female (60.42%), married (84.64%), residing in rural areas (68.87%), with an educational level of primary school or less (77.62%), and a family income less than 15,000/year. The number of participants with cognitive impairments across the four waves of the survey was 662 (67.34%), 708 (72.02%), 729 (74.16%), and 765 (77.82%), respectively. Baseline characteristics of the participants are detailed in Table 1.

**Table 1:** Comparison of basic characteristics of cognitive function in different potential categories of study subjects.

Variable	Trajectory group			Statistic	*p*
	Rapid decline (*n* = 321)	Stable (*n* = 361)	Slow decline (*n* = 301)		
**Age (years)**				48.131	<0.001
45 ~ 59	93 (29.0)	154 (42.7)	170 (56.5)		
≥60	228 (40.3)	207 (36.6)	131 (23.1)		
**Gender, *n* (%)**				20.320	<0.001
Male	95 (29.6)	156 (43.2)	138 (45.8)		
Female	226 (70.4)	205 (56.8)	163 (54.2)		
**Chronic disease, *n* (%)**				0.550	0.760
Yes	271 (84.4)	312 (86.4)	257 (85.4)		
No	50 (15.6)	49 (13.6)	44 (14.6)		
**Smoking**				3.785	0.151
Yes	100 (31.2)	89 (24.7)	88 (29.2)		
No	221 (68.8)	272 (75.3)	213 (70.8)		
**Sleep duration, *n* (%)**				16.844	<0.001
<6 h	173 (53.9)	159 (44.0)	138 (45.8)		
6 ~ 8 h	112 (34.9)	175 (48.5)	143 (47.5)		
>8 h	36 (11.2)	27 (7.5)	20 (6.6)		
**Self-rated health, *n* (%)**				7.230	0.124
Good	24 (7.5)	26 (7.2)	20 (6.6)		
Average	131 (40.8)	166 (46.0)	155 (51.5)		
Poor	166 (51.7)	169 (46.8)	126 (41.9)		
**Hearing ability, *n* (%)**				19.464	<0.001
Good	84 (26.2)	77 (21.3)	101 (33.6)		
Average	156 (48.6)	202 (56.0)	155 (51.5)		
Poor	81 (25.2)	82 (22.7)	45 (15.0)		
**Marital status, *n* (%)**				31.506	<0.001
Married	242 (75.4)	320 (88.6)	270 (89.7)		
Unmarried	79 (24.6)	41 (11.4)	31 (10.3)		
**Residence, *n* (%)**				24.203	<0.001
Rural	242 (75.4)	260 (72.0)	175 (58.1)		
Urban	79 (24.6)	101 (28.0)	126 (41.9)		
**Social participation, *n* (%)**				20.131	<0.001
Yes	154 (48.0)	207 (57.3)	198 (65.8)		
No	167 (52.0)	154 (42.7)	103 (34.2)		
**depression**				25.518	<0.001
Yes	200 (62.3)	207 (57.3)	129 (42.9)		
No	121 (37.7)	154 (42.7)	172 (57.1)		
**Education level, *n* (%)**				172.447	<0.001
Primary school or below	314 (97.8)	284 (78.7)	165 (54.8)		
Middle school	6 (1.9)	57 (15.8)	81 (26.9)		
High school or above	1 (0.3)	20 (5.5)	55 (18.3)		
**Family income, *n* (%)**				48.832	<0.001
<15,000/year	235 (73.2)	220 (60.9)	143 (47.5)		
15,000 ~ 30,000/year	34 (10.6)	38 (10.5)	38 (12.6)		
>30,000/year	52 (16.2)	103 (28.5)	120 (39.9)		
**Medical insurance**				3.438	0.179
Yes	311 (96.9)	357 (98.9)	295 (98.0)		
No	10 (3.1)	4 (1.1)	6 (2.0)		
**Pension insurance**				5.109	0.078
Yes	267 (83.2)	292 (80.9)	229 (76.1)		
No	54 (16.8)	69 (19.1)	72 (23.9)		

### 3.2 Results of the cognitive function LGMM latent class analysis

Models with 1 to 5 classes were set, and the results showed that as the number of classes increased, the values of AIC, BIC, and aBIC decreased, and the Entropy values also changed accordingly. Asparouhov suggested that the number of profiles could be determined by the inflection point where the AIC, BIC, and aBIC values drop (21). Although the AIC, BIC, and aBIC values generally decreased, there was a significant inflection point at the three-class model, where the rate of decline markedly slowed, indicating that the four-class model was not significantly better than the three-class model in terms of AIC, BIC, and aBIC indices. Additionally, the four-class model was merely an extension of the three-class model without providing new theoretical contributions. Howard argued that when profiles have similar theoretical significance, a simpler profile model should be selected (22). Therefore, considering the simplicity of the model, the three-class model was chosen as the optimal model (see Table 2).

**Table 2:** LGMM fitting information of cognitive function change trajectory of study sample.

Model	AIC	BIC	aBIC	Entropy	LMR	BLRT	Class probability
1	25235.513	25264.857	25245.801	–	–	–	–
2	22927.532	22971.547	22942.963	0.908	<0.001	<0.001	0.449/0.550
3	22270.619	22329.306	22291.194	0.866	<0.001	<0.001	0.326/0.367/0.306
4	22016.390	22089.749	22042.109	0.841	<0.001	<0.001	0.227/0.302/0.244/0.227
5	21919.042	22007.073	21949.904	0.838	<0.001	<0.001	0.234/0.235/0.263/0.215/0.051

K, the number of freely estimated parameters; AIC, Akaike Information Guidelines; BIC, Bayesian Information Guidelines; aBIC, Bayesian Information Criteria for Calibration Samples; Entropy, information entropy; LMR, likelihood ratio test; BLRT, a Bootstrap-based likelihood ratio test.

### 3.3 Analysis of latent class trajectories

The estimation results for the three-class LGMM model revealed distinct patterns in cognitive function among the classes: Low-Level Deterioration Group: This group had the lowest average cognitive function score (α = 8.064, *p* < 0.001), with a rapid decline over time (β = −1.571, *p* < 0.001). Normal Aging Group: The cognitive function scores for this group were at a medium level (α = 14.300, *p* < 0.001), showing a gradual decline over time (β = −1.160, *p* < 0.001). High-Level Stability Group: This group had the highest average cognitive function score (α = 18.525, *p* < 0.001), which remained relatively stable (β = −0.150, *p* = 0.164). Graph 2 illustrates the trajectories of cognitive function development for each latent class. Table 3 presents the estimated intercepts and slopes for cognitive function along with their respective statistical test results.

**Table 3:** Intercept and slope estimates of potential categories of cognitive function in the study subjects and their test results.

Class		Estimate	S.E	Est./SE.	*p*-value
Class 1	Intercept(I)	8.064	0.349	23.118	<0.001
	Slope(S)	−1.571	0.095	−16.473	<0.001
Class 2	Intercept(I)	14.300	0.373	38.310	<0.001
	Slope(S)	−1.160	0.126	−9.226	<0.001
Class 3	Intercept(I)	18.525	0.271	68.467	<0.001
	Slope(S)	−0.150	0.108	−1.391	0.164

### 3.4 Univariate analysis of cognition trajectory classes in disabled middle-aged and older adults

Univariate analysis indicated statistically significant differences among the different classes based on age, gender, residence, marital status, education level, family income, sleep duration, hearing ability, depression, and social participation. These results are detailed in Table 1.

### 3.5 Characteristics by cognition of trajectory classes

Using cognitive function trajectory classes as the dependent variable and the variables with statistical significance from univariate analysis as independent variables, a multivariate logistic regression was performed with the “stable” group as the reference. When comparing the “rapid decline” group to the “stable” group, living in rural areas, family income less than 15,000/year, and depression were more likely to be classified as “rapid decline” group. On the other hand, being male, married, having 6–8 h of sleep, social participation, an education level of junior high school and above, and being aged 45–59 were more likely to be classified as “stable” group; When comparing the “slow decline” group to the “stable” group, living in rural areas and depression were more likely to be classified as “slow decline” group. In contrast, having good hearing ability, an education level of junior high school and above, and being aged 45–59 were more likely to be classified as “stable” group. See Table 4 for details.

**Table 4:** Logistic regression analysis of factors influencing cognitive function of research subjects.

Independent variable	Depend variable	β	SE	WaldX^2^	*p*	OR	95%CI
C1 vs. C3	Intercept	1.841	0.523	12.391	<0.001	–	–
	**Age (Reference group: ≥60)**						
	45 ~ 59	−0.853	0.199	18.467	<0.001	0.426	0.289 ~ 0.629
	**Gender (Reference group: Female)**						
	Male	−0.513	0.201	6.546	0.011	0.599	0.404 ~ 0.887
	**Sleep time (Reference group: >8 h)**						
	<6 h	−0.580	0.360	2.595	0.107	0.560	0.277 ~ 1.134
	6 ~ 8 h	−0.747	0.362	4.248	0.039	0.474	0.233 ~ 0.964
	**Hearing ability (Reference group: poor)**						
	Good	−0.218	0.279	0.608	0.435	0.804	0.465 ~ 1.39
	Average	−0.094	0.252	0.140	0.708	0.910	0.556 ~ 1.491
	**Residence (Reference group: Urban)**						
	Rural	0.467	0.212	4.865	0.027	1.595	1.053 ~ 2.415
	**Marital status (Reference group: Unmarried)**						
	Married	−0.859	0.275	9.759	0.002	0.423	0.247 ~ 0.726
	**Social participation (Reference group: No)**						
	Yes	−0.707	0.194	13.267	<0.001	0.493	0.337 ~ 0.722
	**Depression (Reference group: No)**						
	Yes	0.548	0.195	7.877	0.005	1.731	1.180 ~ 2.538
	**Education level (Reference group: Primary school or below)**						
	High school or above	−4.012	1.023	15.391	<0.001	0.018	0.002 ~ 0.134
	Middle school	−2.870	0.444	41.775	<0.001	0.057	0.024 ~ 0.135
	**Family income (Reference group: >30,000)**						
	<15,000	0.714	0.233	9.438	0.002	2.043	1.295 ~ 3.222
	15,000 ~ 30,000	0.340	0.332	1.049	0.306	1.405	0.733 ~ 2.694
C2 vs. C3	intercept	0.731	0.496	2.171	0.141	-	-
	**Age (Reference group: ≥60)**						
	45 ~ 59	−0.488	0.174	7.875	0.005	0.614	0.437 ~ 0.863
	**Gender (Reference group: Female)**						
	Male	−0.084	0.174	0.232	0.630	0.920	0.654 ~ 1.293
	**Sleep time (Reference group: >8 h)**						
	<6 h	−0.295	0.343	0.738	0.390	0.745	0.380 ~ 1.459
	6 ~ 8 h	−0.079	0.340	0.055	0.815	0.924	0.475 ~ 1.798
	**Hearing ability (Reference group: poor)**						
	Good	−0.526	0.257	4.197	0.041	0.591	0.357 ~ 0.977
	Average	−0.079	0.228	0.121	0.728	0.924	0.591 ~ 1.445
	**Residence (Reference group: Urban)**						
	Rural	0.408	0.185	4.875	0.027	1.504	1.047 ~ 2.162
	**Marital status (Reference group: Unmarried)**						
	Married	−0.030	0.276	0.012	0.914	0.971	0.565 ~ 1.666
	**Social participation (Reference group: No)**						
	Yes	−0.289	0.174	2.778	0.096	0.749	0.533 ~ 1.052
	**Depression (Reference group: No)**						
	Yes	0.509	0.173	8.667	0.003	1.664	1.186 ~ 2.337
	**Education level (Reference group: Primary school or below)**						
	High school or above	−1.215	0.296	16.787	<0.001	0.297	0.166 ~ 0.531
	Middle school	−0.748	0.209	12.773	<0.001	0.473	0.314 ~ 0.713
	**Family income (Reference group: >30,000)**						
	<15,000	0.140	0.194	0.518	0.472	1.150	0.786 ~ 1.682
	15,000 ~ 30,000	−0.139	0.283	0.240	0.624	0.870	0.500 ~ 1.516

## 4 Discussion

To the best of our knowledge, this is the first study to analyze the cognitive function trajectories of disabled middle-aged and older adults using data from a 7-year longitudinal study and to explore their predictive factors. Our findings indicate that there are three distinct cognitive function trajectories among disabled middle-aged and older adult individuals: Rapid decline (32.6%), Slow decline (36.1%), and Stable (31.2%).

The management of cognitive function in disabled middle-aged and older adults should focus not only on treatment but also on prevention and control. Therefore, identifying and understanding the cognitive function trajectories and their predictive factors is more meaningful for maintaining cognitive function in this population. Our study results show that disabled middle-aged and older adults who are male, married, have 6–8 h of sleep, good hearing ability, social participation, an education level of junior high school and above, and are middle-aged (45–59 years old) are more likely to maintain a good and stable level of cognitive function. In contrast, those living in rural areas, with family income less than 15,000/year, and with symptoms of depression are more likely to experience a rapid decline in cognitive function. It is evident that the predictive factors of cognitive function trajectories in disabled middle-aged and older adults in China involve not only personal characteristics and behaviors but also interpersonal networks and living and working conditions.

A study on the cognitive trajectories of middle-aged and older adults in China identified three distinct trajectories: slow decline (46.83%), moderate decline (38.44%), and rapid decline (14.73%) (23). Notably, our research found that the proportion of disabled middle-aged and older adults with a rapid cognitive decline trajectory was as high as 32.6%, significantly higher than the rapid decline trajectory in the general older adult population (14.73%). Additionally, even the “stable” group with the highest average cognitive function scores (α = 18.525) exhibited relatively poor cognitive function, indicating that cognitive impairment is more severe among disabled middle-aged and older adults. A study in Japan suggested that physical disability might be associated with neurodegeneration, and the progression of disability is related to cognitive decline (24). For the disabled middle-aged and older adults, it is crucial to establish regular cognitive function assessment mechanisms, as early detection of cognitive issues can facilitate timely intervention measures.

Individual traits, as variables that are not easily changed, are the most direct factors affecting health. Many other factors, such as interpersonal networks, environment, and psychology, influence disease occurrence through their impact on individual traits (20). Middle-aged individuals (45–59 years) are more likely to maintain stable cognitive function compared to older adults (≥60 years), consistent with previous studies (25). As age increases, cognitive abilities tend to decline, which may be related to increased oxidative stress and amyloid protein deposition in the brain leading to brain damage in older adults (26). Men are more likely to maintain stable cognitive function compared to women. This gender difference may be due to differences in socioeconomic status (SES), social networks, engagement in leisure activities, and early life experiences (27, 28).

Behavioral characteristics are closely related to cognitive function in disabled middle-aged and older adults. A national survey indicated that smoking is one of the modifiable risk factors for cognitive impairment (29). In this study, smoking did not have a significant impact on cognitive function in disabled middle-aged and older adults, which may be due to survivor bias among the middle-aged and older adults (30). Disabled middle-aged and older adults who sleep 6–8 h have better cognitive function compared to those with short (<6 h) or long (>8 h) sleep durations, consistent with previous research (31). There is an inverted “U” shaped relationship between sleep duration and the risk of cognitive impairment (32). Short sleep duration (<6 h) may lead to reduced clearance of metabolic waste by the brain’s glymphatic system, chronic neuroinflammation, and hypoxemia (33–35). The decline in cognitive function associated with long sleep duration (>8 h) may be related to increased sleep fragmentation and insufficient deep sleep (36). Since sleep duration is a modifiable and intervenable factor, it is necessary to consider the physical condition, lifestyle, and environmental factors of disabled individuals to develop the most suitable sleep improvement plan. Disabled middle-aged and older adults with good hearing ability are more likely to maintain good cognitive function. Hearing loss has been identified as one of the major modifiable risk factors for dementia (37). The “cascade hypothesis” suggests that hearing loss leads to cognitive decline in a comprehensive manner, where sensory deprivation, social isolation, and increased depressive symptoms due to hearing loss can all contribute to cognitive deterioration (38). For disabled middle-aged and older adults with hearing loss, we recommend the use of hearing aids and cochlear implants to prevent cognitive decline.

Interpersonal networks have a significant impact on the cognitive function of disabled middle-aged and older adults. Those living in urban areas tend to have better cognitive function, as urban regions generally offer more medical resources and health services. Disabled middle-aged and older adults in these areas can more easily access healthcare, rehabilitation services, and treatment, which helps maintain good cognitive function (39). Married disabled middle-aged and older adults also demonstrate better cognitive function, aligning with previous research (40). The resource model suggests that the loss of marriage also means the loss of economic, social, practical, and psychological resources, which are risk factors for cognitive decline (41). Married disabled individuals can receive support and care from their spouses, accompanied by more social interaction and emotional connection, which is beneficial for maintaining cognitive function. Our study shows that disabled middle-aged and older adults with social participation have better cognitive function. Social participation in the disabled population is often overlooked, but our research confirms its value in cognitive function. As one of the three pillars of active aging (42), the cognitive reserve theory proposes that social participation provides mental stimulation through complex communication and interaction with others, which can activate and strengthen various neurobiological pathways, thereby maintaining cognitive function in a relatively stable state (43, 44). Pain, fatigue, and architectural barriers are significant obstacles to the participation of the disabled population (45). Therefore, we recommend providing regular health check-ups and medical services for disabled middle-aged and older adults, and ensuring that public places, residential areas, and transportation facilities are equipped with accessible designs to facilitate social participation. Depression is a predictor of rapid cognitive decline in disabled middle-aged and older adult individuals. Previous research has shown that depressive symptoms precede cognitive decline in the older adult (46). Additionally, depression is a mediating factor for cognitive impairment and functional disability in middle-aged and older adult individuals (47). Our study’s last follow-up was in 2020, during which many COVID-19 survivors experienced persistent depression and related neurocognitive disorders (48, 49). A French study indicated that immune system disorders triggered by COVID-19 infection might induce psychopathology, leading to depressive symptoms and cognitive impairment (50). Some scholars view disability as a chronic stress experience, suggesting that disability interferes with individuals’ perceived social support and sense of control, subsequently increasing the level of depression in the middle-aged and older adults (51). Regular monitoring and detection of depressive symptoms in the disabled population may help slow down irreversible cognitive decline (52).

At the level of work and living environment, our study found that disabled middle-aged and older adults with an education level of junior high school or above had better cognitive abilities. This supports the view that longer education helps to slow down cognitive decline and increases brain cognitive reserve (29). Families income of less than 15,000/year were more likely to fall into the rapid decline group, consistent with previous research (53). Low-income disabled families may face more economic pressure and lack resources, making it difficult for them to obtain adequate medical and healthcare services. In contrast, high-income disabled families may have more resources for a healthy lifestyle and health management, increasing their likelihood of utilizing health services and thereby delaying cognitive decline (54).

The impact of policy environments as distal factors on disabled middle-aged and older adults cannot be ignored. Since 2016, China has implemented long-term care insurance (LTCI) to ensure that disabled individuals can access affordable medical services (55). Research by Ye found that the implementation of LTCI significantly improved the cognitive function of disabled older adults (56). However, our study did not find an impact of medical insurance and pension insurance on the cognitive function trajectories of disabled middle-aged and older adults at the policy environment level. One reason is that our study used data from the 2013 CHARLS for medical and pension insurance. Additionally, 42.4% of our sample were middle-aged individuals (45–59 years old), while long-term care insurance is available from age 60. We recommend formulating future medical policies to support disabled middle-aged individuals and extending the applicable age range for disability benefits to enhance their access to health services.

### 4.1 Strengths and limitations

The main strength of this study is that it is the first to investigate the trajectories of cognitive function changes and their predictors among disabled middle-aged and older adults in China. This research extends the understanding of cognitive function trajectories in the disabled middle-aged and older adults within the socio-economic and cultural context of China. By using a latent growth mixture model, this study overcomes the limitations of traditional growth models that assume all individuals follow the same trajectory. It allows for the identification of distinct subgroups with different trajectory characteristics and trends, which can help in developing targeted intervention and support strategies to improve cognitive function among disabled middle-aged and older adults. Guided by the health ecological model, this study explores the cognitive trajectories and their predictors from factors such as personal characteristics, behavioral lifestyles, and social environments, providing a new perspective for future cognitive function research in the disabled population. Lastly, this study is based on a nationally representative, population-based prospective cohort, making the results highly generalizable and representative.

This study has several limitations. First, we did not categorize the severity of disability. Future research could explore the cognitive change trajectories of middle-aged and older adults with different levels of disability (low, medium, high). Second, due to limitations of the CHARLS database, this study only examined some factors within the health ecological model and did not include physiological and biochemical indicators. Nonetheless, understanding the cognitive function trajectories and their predictors over 7 years in disabled middle-aged and older adults in China remains significant, as it may help the Chinese government in planning and guiding future interventions. Third, the study sample is limited to participants from China, so the generalizability of the findings to other countries or populations remains uncertain. Finally, we used self-reported survey data, which may introduce bias. However, self-reported data are commonly used in disability research of older adults and can more accurately reflect personal status in interaction with the real world (57).

## 5 Conclusion

There are three distinct trajectories of cognitive function in disabled middle-aged and older adults: Rapid decline, Slow decline, and Stable. Preventive measures to address cognitive decline in this population are crucial. We should focus on the value of modifiable factors such as hearing ability, sleep duration, depression, and social participation to achieve healthy aging in disabled middle-aged and older adult individuals.

## Data Availability

The datasets presented in this study can be found in online repositories. The names of the repository/repositories and accession number(s) can be found in the article/supplementary material.

## References

[1] Yao Y Wang K Xiang H. Association between cognitive function and ambient particulate matters in middle-aged and elderly Chinese adults: evidence from the China health and retirement longitudinal study (CHARLS). Sci Total Environ. (2022) 828:154297. doi: 10.1016/j.scitotenv.2022.154297, PMID: 35288137 PMC9112163

[2] Blanco K Salcidua S Orellana P Sauma-Pérez T León T Steinmetz LCL. Systematic review: fluid biomarkers and machine learning methods to improve the diagnosis from mild cognitive impairment to Alzheimer’s disease. Alzheimers Res Ther. (2023) 15:176. doi: 10.1186/s13195-023-01304-8, PMID: 37838690 PMC10576366

[3] Ren R Qi J Lin S Liu X Yin P Wang Z. The China alzheimer report 2022. Gen Psychiatr. (2022) 35:e100751. doi: 10.1136/gpsych-2022-100751, PMID: 35372787 PMC8919463

[4] Khan S Barve KH Kumar MS. Recent advancements in pathogenesis, diagnostics and treatment of Alzheimer’s disease. Curr Neuropharmacol. (2020) 18:1106–25. doi: 10.2174/1570159X18666200528142429, PMID: 32484110 PMC7709159

[5] Zheng X Chen G Song X Liu J Yan L du W. Twenty-year trends in the prevalence of disability in China. Bull World Health Organ. (2011) 89:788–97. doi: 10.2471/BLT.11.089730, PMID: 22084524 PMC3209727

[6] Hanmo Y. Dynamic trend of china’s population ageing and new characteristics of the elderly population research. Popul Res. (2022) 46:104–16.

[7] Brotto D Benvegnù F Colombo A de Filippis C Martini A Favaretto N. Age-related changes in auditory perception. Hearing loss in the elderly: aging ear or aging brain? Aging Clin Exp Res. (2023) 35:2349–54. doi: 10.1007/s40520-023-02570-0, PMID: 37833454 PMC10627897

[8] Mao XY Huang YL Cai QY Wan QR Tang P. Cognitive function and its influencing factors in the disabled elderly in China. Chin J Mult Organ Dis Elder. (2022) 21:91–4. doi: 10.11915/j.issn.1671-5403.2022.02.020

[9] He X Wang X Zhang M Zhu W Liu Y Sun Q. Gender specific cut-off points of age for disability among rural elderly in Anhui Province, China. Front Public Health. (2022) 10:945849. doi: 10.3389/fpubh.2022.945849, PMID: 36268001 PMC9577323

[10] Zhang M Zhu W He X Liu Y Sun Q Ding H. Correlation between functional disability and quality of life among rural elderly in Anhui province, China: a cross-sectional study. BMC Public Health. (2022) 22:397. doi: 10.1186/s12889-021-12363-7, PMID: 35216578 PMC8881859

[11] Li H Li C Wang A Qi Y Feng W Hou C. Associations between social and intellectual activities with cognitive trajectories in Chinese middle-aged and older adults: a nationally representative cohort study. Alzheimers Res Ther. (2020) 12:1–12. doi: 10.1186/s13195-020-00691-6 PMC7519540 32977839

[12] XueMJiaXShiXYangCWangRZhaoC. Association between sarcopenia and cognitive trajectories among middle-aged and older adults in China: a nationally representative cohort study. J Nutr Health Aging. (2023) 27:243–50. doi: 10.1007/s12603-023-1906-137170430

[13] Vaezghasemi M Vogt T Lindkvist M Pulkki-Brännström AM Richter Sundberg L Lundahl L. Multifaceted determinants of social-emotional problems in preschool children in Sweden: an ecological systems theory approach. SSM Popul Health. (2023) 21:101345. doi: 10.1016/j.ssmph.2023.101345, PMID: 36785550 PMC9918800

[14] Liang H Zheng J Sun Y. Prevalence and risk factors associated with circadian syndrome in community-dwelling middle-aged to older adults: based on health ecology model. Sleep Med. (2024) 119:210–3. doi: 10.1016/j.sleep.2024.04.039, PMID: 38703604

[15] Hou D Sun Y Liu Z Sun H Li Y Wang R. A longitudinal study of factors associated with cognitive frailty in middle-aged and elderly population based on the health ecology model. J Affect Disord. (2024) 352:410–8. doi: 10.1016/j.jad.2024.02.014 38367710

[16] Yan Y Du Y Li X Ping W Chang Y. Physical function, ADL, and depressive symptoms in Chinese elderly: evidence from the CHARLS. Front Public Health. (2023) 11:1017689. doi: 10.3389/fpubh.2023.1017689, PMID: 36923048 PMC10010774

[17] Li M Wang N Dupre ME. Association between the self-reported duration and quality of sleep and cognitive function among middle-aged and older adults in China. J Affect Disord. (2022) 304:20–7. doi: 10.1016/j.jad.2022.02.039 35176346

[18] Folstein MF Folstein SE McHugh PR. “Mini-mental state”: a practical method for grading the cognitive state of patients for the clinician. J Psychiatr Res. (1975) 12:189–98. doi: 10.1016/0022-3956(75)90026-6 1202204

[19] Lu J Wang Y Hou L Zuo Z Zhang N Wei A. Multimorbidity patterns in old adults and their associated multi-layered factors: a cross-sectional study. BMC Geriatr. (2021) 21:372. doi: 10.1186/s12877-021-02292-w, PMID: 34147073 PMC8214251

[20] McLeroy KR Bibeau D Steckler A Glanz K. An ecological perspective on health promotion programs. Health Educ Q. (1988) 15:351–77. doi: 10.1177/109019818801500401 3068205

[21] Asparouhov T Muthén B. Auxiliary variables in mixture modeling: three-step approaches using M plus. Struct Equ Model Multidiscip J. (2014) 21:329–41. doi: 10.1080/10705511.2014.915181

[22] Howard J Gagné M Morin AJS van den Broeck A. Motivation profiles at work: a self-determination theory approach. J Vocat Behav. (2016) 95-96:74–89. doi: 10.1016/j.jvb.2016.07.004

[23] Su J Xiao X. Factors leading to the trajectory of cognitive decline in middle-aged and older adults using group-based trajectory modeling: a cohort study. Medicine. (2022) 101:e31817. doi: 10.1097/MD.0000000000031817, PMID: 36451491 PMC9704883

[24] Shimada H Makizako H Doi T Tsutsumimoto K Lee S Suzuki T. Cognitive impairment and disability in older Japanese adults. PLoS One. (2016) 11:e0158720. doi: 10.1371/journal.pone.0158720, PMID: 27415430 PMC4945051

[25] Guo S Zheng XY. New evidence of trends in cognitive function among middle-aged and older adults in China, 2011-2018: an age-period-cohort analysis. BMC Geriatr. (2023) 23:498. doi: 10.1186/s12877-023-04166-9, PMID: 37605117 PMC10440902

[26] Culig L Chu X Bohr VA. Neurogenesis in aging and age-related neurodegenerative diseases. Ageing Res Rev. (2022) 78:101636. doi: 10.1016/j.arr.2022.101636, PMID: 35490966 PMC9168971

[27] Qin Y Liu J Wang R Qi X Jiang S Li J. Can leisure and entertainment lifestyle promote health among older people living alone in China?—a simultaneous equation approach. Front Public Health. (2022) 10:967170. doi: 10.3389/fpubh.2022.967170, PMID: 36249231 PMC9558104

[28] Baiano C Barone P Trojano L Santangelo G. Prevalence and clinical aspects of mild cognitive impairment in Parkinson's disease: a meta-analysis. Mov Disord. (2020) 35:45–54. doi: 10.1002/mds.27902, PMID: 31743500

[29] Jia L Du Y Chu L Zhang Z Li F Lyu D. Prevalence, risk factors, and management of dementia and mild cognitive impairment in adults aged 60 years or older in China: a cross-sectional study. Lancet Public Health. (2020) 5:e661–71. doi: 10.1016/S2468-2667(20)30185-7 33271079

[30] Topiwala A Wang C Ebmeier KP Burgess S Bell S Levey DF. Associations between moderate alcohol consumption, brain iron, and cognition in UK biobank participants: observational and mendelian randomization analyses. PLoS Med. (2022) 19:e1004039. doi: 10.1371/journal.pmed.1004039 35834561 PMC9282660

[31] Hua J Sun H Shen Y. Improvement in sleep duration was associated with higher cognitive function: a new association. Aging (Albany NY). (2020) 12:20623–44. doi: 10.18632/aging.103948, PMID: 33082298 PMC7655193

[32] Bubu OM Brannick M Mortimer J Umasabor-Bubu O Sebastião YV Wen Y. Sleep, cognitive impairment, and Alzheimer’s disease: a systematic review and meta-analysis. Sleep. (2017) 40:zsw032. doi: 10.1093/sleep/zsw032 28364458

[33] Liu H Barthélemy NR Ovod V Bollinger JG He Y Chahin SL. Acute sleep loss decreases CSF-to-blood clearance of Alzheimer's disease biomarkers. Alzheimers Dement. (2023) 19:3055–64. doi: 10.1002/alz.12930, PMID: 36695437 PMC10366339

[34] McAlpine CS Kiss MG Rattik S He S Vassalli A Valet C. Sleep modulates haematopoiesis and protects against atherosclerosis. Nature. (2019) 566:383–7. doi: 10.1038/s41586-019-0948-2, PMID: 30760925 PMC6442744

[35] Qian L Rawashdeh O Kasas L Milne MR Garner N Sankorrakul K. Cholinergic basal forebrain degeneration due to sleep-disordered breathing exacerbates pathology in a mouse model of Alzheimer’s disease. Nat Commun. (2022) 13:6543. doi: 10.1038/s41467-022-33624-y, PMID: 36323689 PMC9630433

[36] Shi L Chen SJ Ma MY Bao YP Han Y Wang YM. Sleep disturbances increase the risk of dementia: a systematic review and meta-analysis. Sleep Med Rev. (2018) 40:4–16. doi: 10.1016/j.smrv.2017.06.010, PMID: 28890168

[37] Livingston G Sommerlad A Orgeta V Costafreda SG Huntley J Ames D. Dementia prevention, intervention, and care. Lancet. (2017) 390:2673–734. doi: 10.1016/S0140-6736(17)31363-6 28735855

[38] Denham MW Weitzman RE Golub JS. Hearing aids and cochlear implants in the prevention of cognitive decline and dementia—breaking through the silence. JAMA Neurol. (2023) 80:127–8. doi: 10.1001/jamaneurol.2022.4155 36469311

[39] Pan Y Wu X Liu Y Li Z Yang Y Luo Y. Urbanization and cognitive function among middle-aged and old adults in China. J Gerontol B Psychol Sci Soc Sci. (2022) 77:2338–47. doi: 10.1093/geronb/gbac102 35908238

[40] Liu H Zhang Y Burgard SA Needham BL. Marital status and cognitive impairment in the United States: evidence from the National Health and aging trends study. Ann Epidemiol. (2019) 38:e2:28–34.e2. doi: 10.1016/j.annepidem.2019.08.007 PMC6812624 31591027

[41] Wu-Chung EL Leal SL Denny BT Cheng SL Fagundes CP. Spousal caregiving, widowhood, and cognition: a systematic review and a biopsychosocial framework for understanding the relationship between interpersonal losses and dementia risk in older adulthood. Neurosci Biobehav Rev. (2022) 134:104487. doi: 10.1016/j.neubiorev.2021.12.010, PMID: 34971701 PMC8925984

[42] Nie Y Richards M Kubinova R Titarenko A Malyutina S Kozela M. Social networks and cognitive function in older adults: findings from the HAPIEE study. BMC Geriatr. (2021) 21:1–14. doi: 10.1186/s12877-021-02531-0 34663241 PMC8524850

[43] Fratiglioni L Wang HX Ericsson K Maytan M Winblad B. Influence of social network on occurrence of dementia: a community-based longitudinal study. Lancet. (2000) 355:1315–9. doi: 10.1016/S0140-6736(00)02113-9 10776744

[44] Li G Larson EB Shofer JB Crane PK Gibbons LE McCormick W. Cognitive trajectory changes over 20 years before dementia diagnosis: a large cohort study. J Am Geriatr Soc. (2017) 65:2627–33. doi: 10.1111/jgs.15077, PMID: 28940184 PMC5729097

[45] Heath GNV Fentem PH. 8 physical activity among persons with disabilities—a public health perspective. Exerc Sport Sci Rev. (1997) 25:195–234. 9213093

[46] Yuan J Wang Y Liu Z. Temporal relationship between depression and cognitive decline in the elderly: a two-wave cross-lagged study in a Chinese sample. Aging Ment Health. (2023) 27:2179–86. doi: 10.1080/13607863.2023.2225432, PMID: 37339082

[47] Liu M Du X Sun Y Zhou A Sun S Wu Y. The mediating role of cognition in the relationship between sleep duration and instrumental activities of daily living disability among middle-aged and older Chinese. Arch Gerontol Geriatr. (2021) 94:104369. doi: 10.1016/j.archger.2021.104369, PMID: 33556636

[48] Lu J Xu X Huang Y Li T Ma C Xu G. Prevalence of depressive disorders and treatment in China: a cross-sectional epidemiological study. Lancet Psychiatry. (2021) 8:981–90. doi: 10.1016/S2215-0366(21)00251-0, PMID: 34559991

[49] Mazza MG De Lorenzo R Conte C Poletti S Vai B Bollettini I. Anxiety and depression in COVID-19 survivors: role of inflammatory and clinical predictors. Brain Behav Immun. (2020) 89:594–600. doi: 10.1016/j.bbi.2020.07.037, PMID: 32738287 PMC7390748

[50] Mazza MG Palladini M De Lorenzo R Magnaghi C Poletti S Furlan R. Persistent psychopathology and neurocognitive impairment in COVID-19 survivors: effect of inflammatory biomarkers at three-month follow-up. Brain Behav Immun. (2021) 94:138–47. doi: 10.1016/j.bbi.2021.02.021, PMID: 33639239 PMC7903920

[51] Tian G Li R Cui Y Zhou T Shi Y Yang W. Association between disability, social support and depressive symptoms in Chinese older adults: a national study. Front Public Health. (2022) 10:980465. doi: 10.3389/fpubh.2022.980465, PMID: 36062100 PMC9437525

[52] Wang J Liang X Qiu Q Yan F Fang Y Shen C. Cognitive trajectories in older adults and the role of depressive symptoms: a 7-year follow-up study. Asian J Psychiatr. (2024) 95:104007. doi: 10.1016/j.ajp.2024.104007, PMID: 38520944

[53] Chen L Cao Q. Poverty increases the risk of incident cognitive impairment among older adults: a longitudinal study in China. Aging Ment Health. (2020) 24:1822–7. doi: 10.1080/13607863.2019.1663491, PMID: 31496262

[54] Chen X Giles J Yao Y Yip W Meng Q Berkman L. The path to healthy ageing in China: a Peking University–lancet commission. Lancet. (2022) 400:1967–2006. doi: 10.1016/S0140-6736(22)01546-X, PMID: 36423650 PMC9801271

[55] Li Q Chen Y Zhang Y Liu X. Evaluation of China’s long-term care insurance policies. Front Public Health. (2024) 12:1252817. doi: 10.3389/fpubh.2024.1252817, PMID: 38605882 PMC11007106

[56] Ye X Hu M Lin H. Effects of the long-term care insurance on health among older adults: a panel data from China. Int J Health Policy Manag. (2023) 12:7664. doi: 10.34172/ijhpm.2023.7664 38618818 PMC10590242

[57] Smith KV Goldman N. Measuring health status: self-, interviewer, and physician reports of overall health. J Aging Health. (2011) 23:242–66. doi: 10.1177/0898264310383421, PMID: 21041293 PMC3727648

